# Triglyceride glucose index is associated with obstructive coronary artery disease in hypertensive patients

**DOI:** 10.1186/s12933-023-01739-1

**Published:** 2023-01-12

**Authors:** Weili Pan, Yongkui Ren, Fan Yang, Minxian Wang, Xinsheng Li, Da Yin

**Affiliations:** 1 Department of Cardiology, the First Affiliated Hospital of Dalian Medical University, Dalian, Liaoning China; 2 Department of Cardiology, the Affiliated Zhongshan Hospital of Dalian University, Dalian, Liaoning China; 3 Department of Cardiology, Shenzhen People’s Hospital, 2nd Clinical Medical College of JINAN University, 1st Affiliated Hospital of the Southern University of Science and Technology. No. 1017 Dongmen North Road, Luohu District, Shenzhen, China

**Keywords:** TyG index, Obstructive CAD, Hypertensive patients

## Abstract

**Background:**

Hypertension is a leading risk of coronary artery disease (CAD). Triglyceride glucose index (TyG) is a surrogate of insulin resistance (IR). Few studies explore the association between TyG and the incidence of obstructive CAD (OCAD) in hypertensive patients.

**Methods:**

We retrospectively screened 1841 hypertensive subjects who were free of a history of CAD and underwent coronary computed tomography angiography (CCTA) because of chest pain. TyG index was calculated as ln (fasting TG [mg/dL] * fasting glucose [mg/dL]/2). The outcome of this research was OCAD, which was defined as the presence of diameter stenosis ≥ 50% in any of the four major epicardial coronary arteries detected on CCTA.

**Results:**

A total of 310 (16.8%) patients developed obstructive CAD. The restricted cubic spline (RCS) analysis showed a J-shaped relationship between TyG and OCAD and the OR for OCAD increased as the TyG rose over 8.61 (OR perSD) 1.64, 95% CI 1.13–2.54, *p* = 0.008). After full adjustments for confounding covariates, patients with TyG index in tertile 3 (T3) had 2.12 times (95% CI 1.80 to 3.81) and in T2 had 2.01 times (95% CI 1.40 to 2.88) as high as the risk of OCAD compared with patients in T1 (*p* for trend = 0.001). When regarding TyG as a continuous variable, 1-SD increase elevated 49% (OR (95%CI), 1.49 (1.30–1.74)) risk of obstructive CAD (*p* = 0.007). This positive effect was still consistent across the subgroups (*p* for interaction > 0.05).

**Conclusion:**

TyG index was associated with the incidence of obstructive CAD in hypertensive patients.

## Introduction

Approximately 31.1% adults are affected by hypertension worldwide and the figure surges globally with the aging of the population [1]. Hypertension, as the leading risk factor for atherosclerosis, leads to 47% of ischemic heart disease [2]. Hypertensive individuals are at high risk of coronary artery disease (CAD). However, a large proportion of patients do not have CAD. Thus, it is unreasonable and cost-inefficient to prescript coronary computed tomography angiography (CCTA) to all hypertensive patients. Given the associations between the two factors and the risks related to missed diagnoses, it is arguably necessary for cardiologists to risk stratify hypertensive individuals and guide further examinations.

Triglyceride glucose index (TyG), which is calculated from fasting glucose and triglycerides, has been regarded as an inexpensive surrogate of insulin resistance (IR) in the setting of clinical practice, and even shows better performance compared with the traditional homeostasis model (HOMA-IR) in evaluating IR [3, 4]. Accumulated evidence has revealed that the TyG index is closely related to cardiovascular diseases, including carotid atherosclerosis, metabolic syndrome, subclinical atherosclerosis, and coronary artery calcification [5–8]. Furthermore, an increased TyG index not only is associated with the incidence of atherosclerotic disease but also can predict adverse outcomes in patients with established CAD [9, 10]. Generally, some advances about the potential roles of TyG in cardiovascular diseases have been made. However, relevant researchers, focusing on the relations of the TyG with the prevalence of obstructive coronary artery disease (OCAD) in patients with hypertension, are still lacking.

The present study aimed to investigate the relationships between TyG and the incidence of obstructive CAD in hypertensive patients.

## Methods

### Study population and data definition

**Fig. 1:**
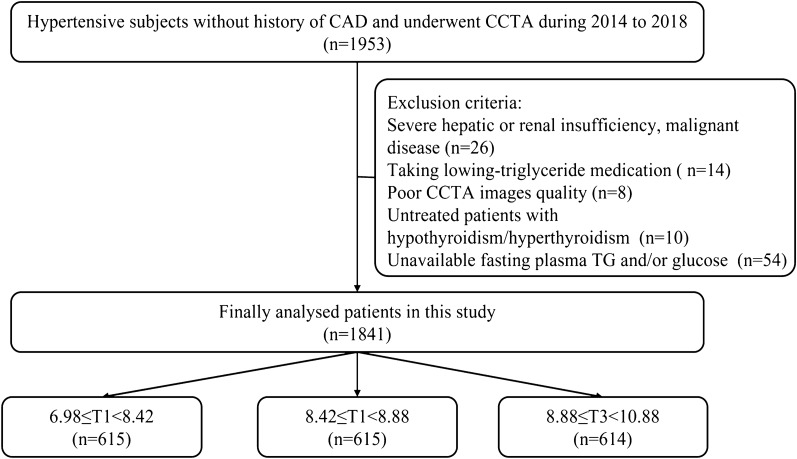
Study flow chart. CAD, coronary artery disease; CCTA, coronary computed tomography angiography; TG, triglyceride T, tertile

This was an observational cross-sectional study. We retrospectively screened 1953 hypertensive subjects who were free of history of CAD and underwent coronary computed tomography angiography (CCTA) because of typical/atypical chest pain in the Department of Hypertension in the First Affiliated Hospital of Dalian Medical University from January 2014 to December 2018. The detailed study flow chart was shown in Fig. 1. Hypertension was defined as a prior diagnosis of hypertension or the use of antihypertensive medications or newly diagnosed hypertension according to the 2017 ACC/AHA guideline (systolic BP ≥ 140 mm Hg and/or diastolic BP ≥ 90 mm Hg). Patients previously diagnosed with CAD according to CCTA, coronary angiography, or treadmill exercise testing weren’t included in the present study. Data of demographic characteristics, previous medical history, laboratory examinations, coronary artery calcification score (CACS), and imaging parameters were analyzed. Laboratory parameters were from fasting venous blood and detected in the biochemistry lab of the First Affiliated Hospital of Dalian Medical University. Moreover, the calculation of LDL cholesterol (LDL-C) was done using the Friedewald equation. Patients with severe hepatic/renal insufficiency, malignant disease, poor CCTA image quality, untreated hyperthyroidism/hypothyroidism, and unavailable fasting glucose and triglyceride (TG) levels were excluded. Furthermore, patients taking lowing-triglyceride medication were not included and 1841 available subjects were finally analyzed in the study (a detailed flow chart was shown in Fig. 1). Specifically, the TyG index was calculated as ln (fasting TG [mg/dL] * fasting glucose [mg/dL]/2).

This study was approved by the First Affiliated Hospital of Dalian Medical University Ethics Committee.

### Coronary computed tomography angiography and coronary artery calcium scanning

According to the guidelines outlined by the Society of Cardiovascular Computed Tomography, CCTA image acquisition, and processing, as well as coronary artery calcium scanning, were performed on the scanner (dual-source, Somatom Definition CT, Siemens, Erlangen, Germany). Two professional imaging physicians blind to the patient's clinical data independently evaluated all images to determine the extent of CAD and provide a CACS using the Agatston method which semi-automatically calculates a weighted sum of the area of coronary calcification in line with the available study [11]. The outcome of the present research was obstructive CAD (OCAD), which was defined as the presence of diameter stenosis ≥ 50% in any of the four major epicardial coronary arteries detected on CCTA.

### Statistical analysis

Normally distributed continuous variables were expressed as mean ± standard deviation (SD) and compared with the t-test; non-normally distributed continuous variables were expressed as median (interquartile range (IQR)) and compared with the non-parametric test. Chi-square tests or Fisher’s exact test was used to assess the differences between categorical variables, which are reported as a number (percentage).

Restricted cubic spline (RCS) analysis was performed to detect the non-linear relationships between the TyG index and the prevalence of obstructive CAD. A univariable logistic regression analysis was performed to detect the association between variables and obstructive CAD and variables with *p* < 0.1 (except for triglyceride (TG) because of collinearity with TyG index) were adjusted in the following multivariable analysis. A multivariable logistic regression analysis with backward stepwise selection was applied to verify the independent associations between TyG and the incidence of obstructive CAD and related results were reported as odds ratios (ORs) and 95% confidence intervals (CIs). In model 1, age, sex, history of diabetes mellitus (DM), and current smoker were adjusted; in model 2, high-density lipoprotein cholesterol (HDL-C), high sensitivity C-reaction protein (Hs-CRP), and lipoprotein A (Lpa) were further adjusted based on model 1. Additional E/e’ was adjusted in model 3. Moreover, the logistic regression results were represented according to TyG tertiles (as a categorical variable) using the lowest tertile 1 (T1) as the reference and by per SD increase in TyG (as a continuous variable) in the interpretation of results. Furthermore, stratified analyses by sex, age > 50, history of type 2 DM, and CACS > 100U were shown in a fully adjusted model and their interactions were detected. ORs and CI s, and *p* for interaction were represented in the interpretation of results.

A two-tailed *p*-value of < 0.05 was considered statistically significant in the present study. All of the analyses were performed with the statistical software R V.4.1.0 (R Foundation for Statistical Computing, Vienna, Austria).

## Results

### Baseline characteristics of the patient population

A total of 1841 available patients with hypertension were finally analyzed in this study, which was consisted of 54.3% males and 26.1% patients with diabetes. A total of 310 (16.8%) patients developed obstructive CAD. The mean age of the study population was 58.3 ± 14.0 years. The median TyG index was 8.61 (IQR 8.28–9.05). Detailed characteristics of the study population were shown in Table 1. Available subjects were divided into three groups according to the TyG index tertile. Patients with the highest TyG index were more likely to be younger, male, current smokers, and have a history of DM (all the *p* < 0.05). TyG index seemed to be positively related with TC, TG, LDL-C, Hs-CRP, HBA1C, fasting glucose, creatinine, E/e’, and CACS, whereas it was inversely associated with HDL-C and Lpa (all the *p* < 0.05). Moreover, the prevalence of obstructive CAD markedly increased with the increasing of TyG index (*p* < 0.001).

**Table 1:** Demographic characteristics of the study population

Variables	TyG index level				*p*
	Total (n = 1841)	(6.98 ≤ T1 < 8.42) (n = 615)	(8.42 ≤ T2 < 8.88) (n = 612)	(8.88 ≤ T3 < 10.88) (*n* = 614)	
Age, years	58.3 ± 14.0	59.7 ± 14.3	58.0 ± 14.1	57.3 ± 13.7	0.010
Male, n (%)	999 (54.3%)	303 (49.3%)	349 (57.0%)	347 (56.5%)	0.009
Duration of hypertension, years	10.2 ± 8.4	9.3 ± 8.0	10.1 ± 8.3	10.3 ± 7.9	0.024
Newly diagnosed hypertension, n (%)	125(6.8%)	36 (5.9%)	41 (6.7%)	48(7.8%)	0.227
*Cardiovascular risks*					
Current smoker, n (%)	520 (28.2%)	146 (23.7%)	179 (29.2%)	195 (31.8%)	0.006
Diabetes mellitus, n (%)	480 (26.1%)	72 (11.7%)	143 (23.4%)	265 (43.2%)	< 0.001
*Baseline laboratory data*					
TC, mmol/L	4.76 ± 1.03	4.45 ± 0.97	4.83 ± 0.98	5.01 ± 1.05	< 0.001
TG, mmol/L	1.63 ± 0.96	0.91 ± 0.22	1.43 ± 0.27	2.56 ± 1.11	< 0.001
HDL-C, mmol/L	1.14 ± 0.28	1.25 ± 0.31	1.12 ± 0.25	1.04 ± 0.24	< 0.001
LDL-C, mmol/L	2.61 ± 0.71	2.37 ± 0.66	2.70 ± 0.68	2.78 ± 0.73	< 0.001
Hs-CRP, mmol/L	1.00 (0.54–2.05)	0.79 (0.39–1.50)	1.01 (0.58–1.96)	1.08 (0.65–2.71)	< 0.001
Lpa, mg/L	144 (76.0–265)	152 (85.0–299)	147 (85.0–259)	118 (61.0–234)	< 0.001
HbA1C, %	6.12 ± 0.99	5.74 ± 0.45	6.05 ± 0.85	6.56 ± 1.30	< 0.001
Creatinine, μmmol/L	65.0 (55.0–77.0)	65.0 (54.0–75.0)	65.0 (56.0–79.0)	66.0 (54.0–77.0)	0.018
LVEF	58.0 ± 4.65	58.0 ± 4.81	57.9 ± 4.93	58.1 ± 4.17	0.663
E/e’	8.88 ± 3.19	8.65 ± 3.32	8.86 ± 3.07	9.11 ± 3.17	0.040
CACS	3.70 (0.00–119)	0.70 (0.00–78.6)	2.75 (0.00–126)	8.70 (0.00–172)	0.011
*Outcome*					
OCAD, n (%)	310 (16.8%)	62 (10.1%)	111 (18.1%)	137 (22.3%)	< 0.001

Data are presented as mean ± SD, median [25th–75th percentiles] or n ± %)

TyG, triglyceride glucose index; TC, Total cholesterol; TG, Triglycerides; HDL-C, high density lipoprotein cholesterol; LDL-C, low density lipoprotein cholesterol; Hs-CRP, high sensitivity C-reaction protein, LVEF, left ventricular ejection fraction; CACS, coronary artery calcium score; OCAD, obstructive coronary heart disease

### Relationship between the TyG index and incidence of obstructive CAD in hypertensive patients

**Fig. 2:**
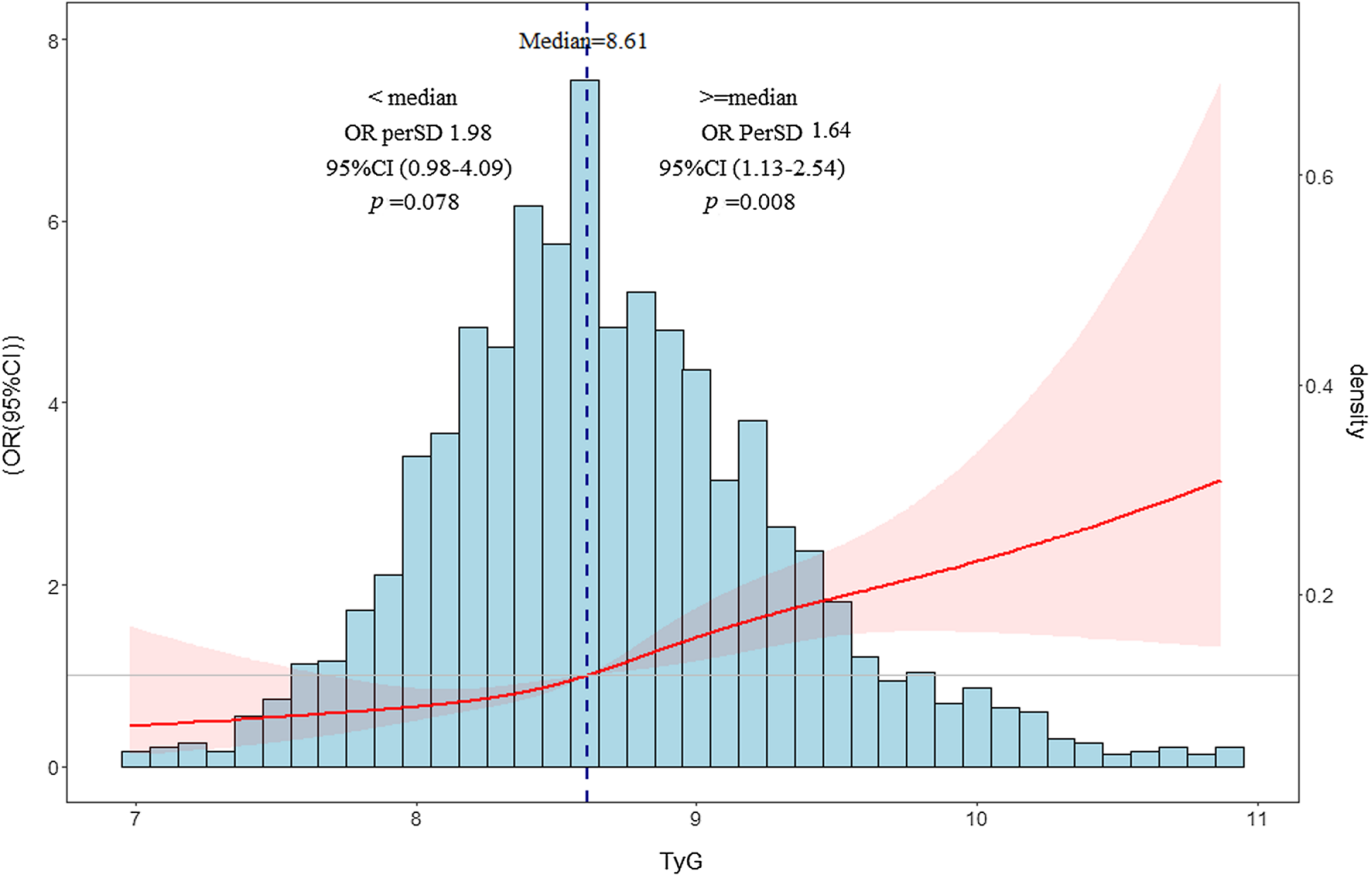
The restricted cubic spline of TyG index and the incidence of obstructive CAD. The blue columns present the distribution density of the TyG index. The grey line represents the OR = 1. The red line shows the OR value. The red shaded area represents the 95% CI. OR, odds ratio, CI confidence interval, CAD, coronary artery disease; TyG index, triglyceride glucose index; RCS, restricted cubic spline

To visualize the non-linear relationship between the TyG index and the occurrence of obstructive CAD, RCS analysis was applied (*p* for nonlinear = 0.354). As shown in Fig. 2, the RCS curve appeared to be J-shaped. For the TyG index < 8.61, the OR per SD was 1.98 (95% CI 0.98–4.09, *p* = 0.078). However, with the TyG index increased over 8.61, the OR for the prevalence of obstructive CAD significantly increased (OR per SD 1.64, 95% CI 1.13–2.54, *p* = 0.008).

In univariable analysis (as shown in Table 2), TyG (T2) and TyG (T3) were markedly related to the risk of obstructive CAD compared with the reference group (OR (95%CI) 1.976 (1.416–2.758) and 2.562 (1.853–3.542), respectively). A similar result was found in TyG PerSD increase (OR 1.46; 95% CI 1.29 to 1.65).

**Table 2:** Univariate logistic regression for the prevalence of CHD in hypertensive patients

Variables	OR (95%CI)	*p* value
Age	1.05 (1.04–1.06)	< 0.001
gender	1.64 (1.27–2.11)	< 0.001
Duration of hypertension	0.89 (0.44–1.36)	0.658
DM	2.44 (1.89–3.15)	< 0.001
Current smoker	2.15 (1.67–2.77)	< 0.001
Hs-CRP	1.01 (0.99–1.02)	0.081
LDL-C	1.03 (0.86–1.22)	0.777
HDL-C	0.35 (0.22–0.56)	< 0.001
TC	0.99 (0.88–1.11)	0.853
TG	1.25 (1.12–1.40)	< 0.001
CRE	0.99 (0.98–1.00)	0.387
LPa	1.00 (1.00–1.01)	0.06
LVEF	0.99 (0.97–1.02)	0.438
E/e’	1.07 (1.04–1.11)	< 0.001
TyG(T2)	1.98 (1.42–2.76)	< 0.001
TyG(T3)	2.56 (1.85–3.54)	< 0.001
TyG(PerSD)	1.46 (1.29–1.65)	< 0.001

Data are presented as OR (95%CI)

TC, Total cholesterol; TG, Triglycerides; eGFR, HDL-C, high density lipoprotein cholesterol; LDL-C, low density lipoprotein cholesterol; Hs-CRP, high sensitivity C-reaction protein; LVEF, left ventricular ejection fraction; CACS, coronary artery calcium score; CHD, coronary heart disease; TyG, tyiglyceride glucose index

**Fig. 3:**
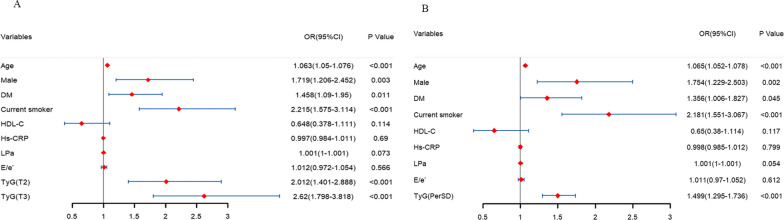
Risk of obstructive CAD based on model 3. TyG index was regarded as a categorical variable in **A** and as a continuous variable in **B**. Model 3 was adjusted for age, gender, history of diabetes, current smoker, HDL-C, Hs-CPR, Lpa, and E/e’. TyG, triglyceride glucose index; HDL-C, high-density lipoprotein cholesterol; Hs-CRP, high sensitivity C-reaction protein, Lpa, lipoprotein a; CHD, coronary heart disease. 6.98 ≤ T1 < 8.42; 8.42 ≤ T2 < 8.88; 8.88 ≤ T3 < 10.88

As shown in Table 3 and Fig. 3A, the risk of obstructive CAD significantly rised as the TyG index tertiles increased in the unadjusted model, Model 1, Model 2, and Model 3 (*p* for trend < 0.001, < 0.001, = 0.001, and = 0.001, respectively). Specifically, in Model 1, a 2.12-fold increased risk of obstructive CAD for patients with TyG values in T2 (OR 2.12; 95%CI 1.49 to 3.05) and a 2.78-fold increased risk for patients with TyG values in T3 (OR 2.78; 95%CI 1.94 to 4.02) was observed compared with the TyG (T1). When HDL-C, TG, Hs-CPR, Lpa, and E/e’ were further adjusted in Model 2 and Model 3, TyG still remained related to more than a twofold increased risk of obstructive CAD. When analyzing the TyG index as a continuous variable (as shown in Fig. 3B), a 1-SD increase in TyG was associated with an almost 50% elevated risk of obstructive CAD in hypertensive patients in four models (all the *p* < 0.05).

**Table 3:** Association between TyG index and CHD in hypertensive patients

	TyG(T1)	TyG(T2)	TyG(T3)	*p* for trend	1-SD increase in TyG
*Unadjusted*				< 0.001	
OR (95%CI)	1 (reference)	1.97 (1.42–2.76)	2.56 (1.85–3.54)		1.46 (1.29–1.65)
*p*. value		< 0.001	< 0.001		< 0.001
*Model 1*				< 0.001	
OR (95%CI)	1 (reference)	2.12 (1.49–3.05)	2.78 (1.94–4.02)		1.53 (1.33–1.76)
*p*. value 1		< 0.001	< 0.001		< 0.001
*Model 2*				0.001	
OR (95%CI)	1 (reference)	2.01 (1.41–2.90)	2.62 (1.81–3.84)		1.47(1.12–1.94)
*p*. value 2		0.002	0.01		0.006
*Model 3*				0.001	
OR (95%CI)	1 (reference)	2.01 (1.40–2.88)	2.63 (1.80–3.81)		1.49 (1.30–1.74)
*p*. value 3		0.001	0.001		0.007

Model 1: adjusted for age, gender, diabetes and current smoker

Model 2: further adjusted for HDL-C, Hs-CPR, Lpa

Model 3: Model 2 + E/e’

TyG, triglyceride glucose index; TG, Triglycerides; HDL-C, high density lipoprotein cholesterol; Hs-CRP, high sensitivity C-reaction protein, CHD, coronary heart disease

**Fig. 4:**
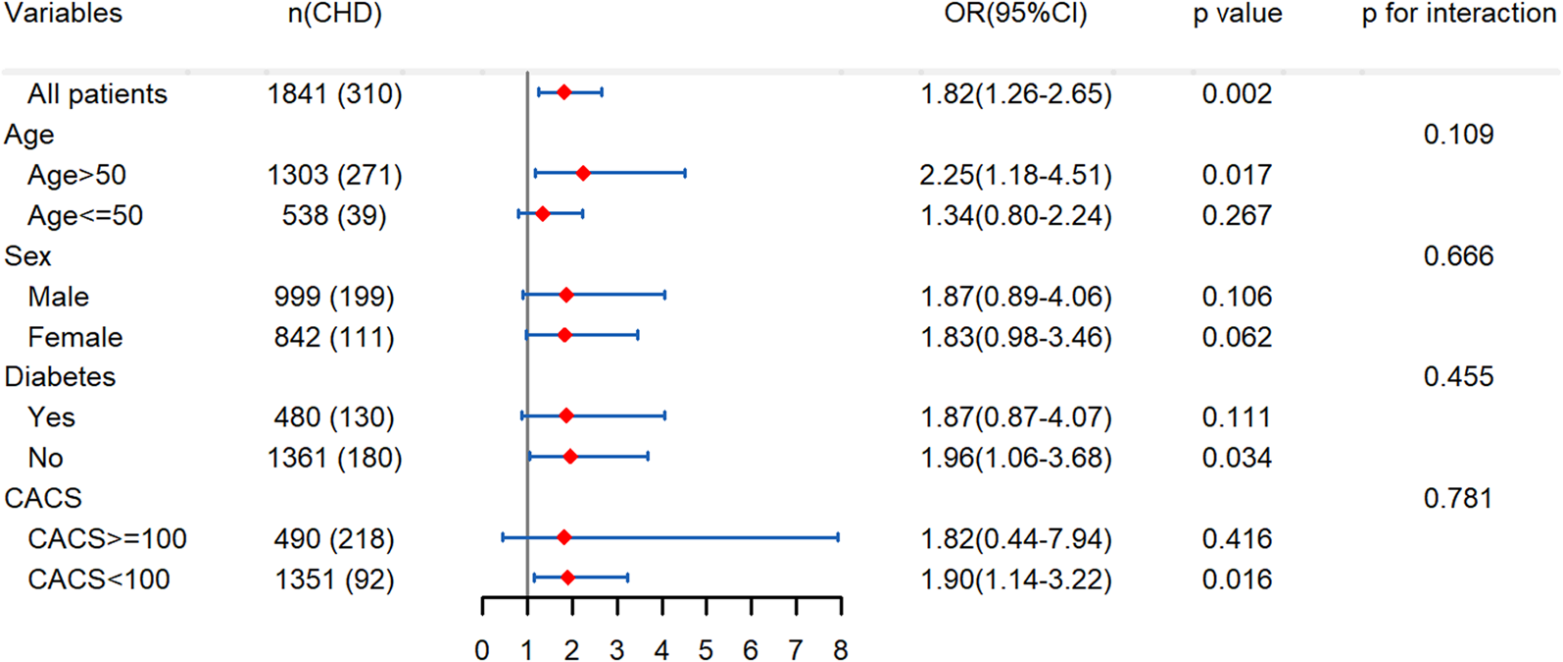
Stratified analysis for the association of TyG with obstructive CAD based on model 3. Model 3 was adjusted for age, gender, history of diabetes, current smoker, HDL-C, Hs-CPR, Lpa, and E/e′. TyG, triglyceride glucose index; HDL-C, high-density lipoprotein cholesterol; Hs-CRP, high sensitivity C-reaction protein, Lpa, lipoprotein a; CHD, coronary heart disease

Results from sensitivity analyses were shown in Fig. 4. The positive effect of TyG on the occurrence of obstructive CAD was consistent across the subgroups after adjustments for the confounding factors. *P* for interaction was 0.109, 0.666, 0.455, and 0.781 for age, sex, DM, and CACS subgroups, respectively.

## Discussion

In the present study, the TyG index has a significant and positive effect on the prevalence of obstructive CAD in hypertensive patients. This demonstrates that the TyG index may be useful in identifying patients with hypertension at high risk of obstructive CAD and guiding further detections and more aggressive treatments.

High blood pressure is one of the leading risk factors worldwide, which is obligated to 90% of the attributable risk for myocardial infarction in men and 94% in women [12]. Thus, it is necessary to identify hypertensive subjects at high risk for CAD to guide further detection and early therapies. To the best of our knowledge, our study is the first study to reveal the graded associations between the TyG index and the incidence of obstructive CAD in hypertensive patients. This result was upheld by some previous researchers in some ways. A larger-scale study with 59,123 participants has revealed that the TyG index is positively related to the development and severity of carotid atherosclerosis assessed by ultrasonography [13]. Moreover, recent researches have also shown that the TyG index is a significant predictor for the progression of coronary atherosclerosis in patients with established CAD irrespective of other risk factors, and of coronary calcification in individuals free of the previous CAD [8, 14]. Furthermore, a body of accumulating evidence has demonstrated that the TyG index can predict the occurrence of major adverse cardiovascular events regardless of diabetes status [15–19]. In addition, we have noted that earlier researchers have reached similar conclusions. The TyG index is an available predictor for the severity of CAD in patients with established CAD [20, 21], regardless of glucose metabolic states. Meanwhile, this conclusion is also tenable in individuals with non-alcoholic fatty liver disease and type 2 diabetes [22, 23]. Nevertheless, compared with previous evidence, the present study focused on hypertensive subjects free of a history of CAD and revealed that the TyG index was associated with obstructive CAD in hypertensive patients, which has filled some gaps in the field of the relationships between the TyG index and occurrence of CAD.

TyG index is a practical surrogate of insulin resistance and even shows better performance compared with the traditional homeostasis model (HOMA-IR) in evaluating IR. Insulin resistance has been reported to be a pivotal risk for the development and progression of CAD. Theoretically, insulin resistance could result in endothelial dysfunction, coagulation dysfunction, oxidative stress, and inflammation [24]. Admittedly, these pathological processes are widely involved in the development and progression of CAD. Furthermore, insulin resistance is tightly related to the acknowledged coronary disease hazard factors, including obesity, hyperinsulinemia, hypertension, hyperlipidemia, and diabetes mellitus [25]. Not surprisingly, as represented in the present study, the TyG index was qualified to predict the prevalence of obstructive CAD in patients with hypertension.

Patients with hypertension are a special group, who are usually complicated by multiple metabolic disorders, including diabetes, obesity, hyperlipidemia, and so on. Moreover, it is acknowledged that TyG is positively associated with the prevalence of type 2 diabetes, hypertension, and coronary calcification [8, 26]. As the same results in the present study, the incidence of type 2 diabetes and CACS increased with the increasing of the TyG index. Furthermore, subgroup analysis is performed to validate the universality of the conclusions. Our research has demonstrated TyG index can predict the occurrence of obstructive CAD in patients with hypertension, irrespective of age, sex, diabetes state, and CACS. In other words, the conclusion was extensively practicable in hypertensive patients.

There are some limitations of this study. Firstly, this is a retrospective observational cross-sectional study. We consecutively screened subjects. However, some inherent deviations are subsistent. Secondly, obesity, as an important risk factor for cardiovascular diseases, was not adjusted in the current study. Thirdly, it is reported that the consistency between CCTA and coronary angiography is well. Nevertheless, CCTA is still not the gold standard for access severity of coronary stenosis, due to the accuracy of CCTA may be affected by severe coronary calcification. Hence, more than 50% stenosis on CCTA may not represent the accuracy > 50% stenosis evaluated by coronary angiography. Fourthly, further follow-up is necessary.

## Conclusion

Our study provided some evidence that the TyG index was related to the incidence of obstructive CAD in hypertensive patients, irrespective of established clinical risks. This will provide valuable information for early risk stratification and guiding further detections and more aggressive treatments.

## Data Availability

The data are available from the corresponding authors upon reasonable request.
